# Modeling Predictability of Traffic Counts at Signalised Intersections Using Hurst Exponent

**DOI:** 10.3390/e23020188

**Published:** 2021-02-03

**Authors:** Sai Chand

**Affiliations:** Research Centre for Integrated Transport Innovation (rCITI), School of Civil and Environmental Engineering, University of New South Wales, Sydney, NSW 2052, Australia; saichand.chakka@unsw.edu.au

**Keywords:** intersections, traffic count, predictability, Hurst exponent

## Abstract

Predictability is important in decision-making in many fields, including transport. The ill-predictability of time-varying processes poses severe problems for traffic and transport planners. The sources of ill-predictability in traffic phenomena could be due to uncertainty and incompleteness of data and models and/or due to the complexity of the processes itself. Traffic counts at intersections are typically consistent and repetitive on the one hand and yet can be less predictable on the other hand, in which on any given time, unusual circumstances such as crashes and adverse weather can dramatically change the traffic condition. Understanding the various causes of high/low predictability in traffic counts is essential for better predictions and the choice of prediction methods. Here, we utilise the Hurst exponent metric from the fractal theory to quantify fluctuations and evaluate the predictability of intersection approach volumes. Data collected from 37 intersections in Sydney, Australia for one year are used. Further, we develop a random-effects linear regression model to quantify the effect of factors such as the day of the week, special event days, public holidays, rainfall, temperature, bus stops, and parking lanes on the predictability of traffic counts. We find that the theoretical predictability of traffic counts at signalised intersections is upwards of 0.80 (i.e., 80%) for most of the days, and the predictability is strongly associated with the day of the week. Public holidays, special event days, and weekends are better predictable than typical weekdays. Rainfall decreases predictability, and intersections with more parking spaces are highly predictable.

## 1. Introduction

Signalised intersections play a crucial role in improving the performance of urban road networks. Statistical data indicate that two-thirds of urban vehicle miles travelled in the US are on signal-controlled roadways [1]. Ensuring the efficient operation of traffic signals can minimise vehicle delays, maximise vehicle throughput, and improve air quality to serve the ever-rising travel demand. To achieve these goals, understanding the temporally varying patterns of demand and intersection vehicle throughput is essential.

**Figure 1:**
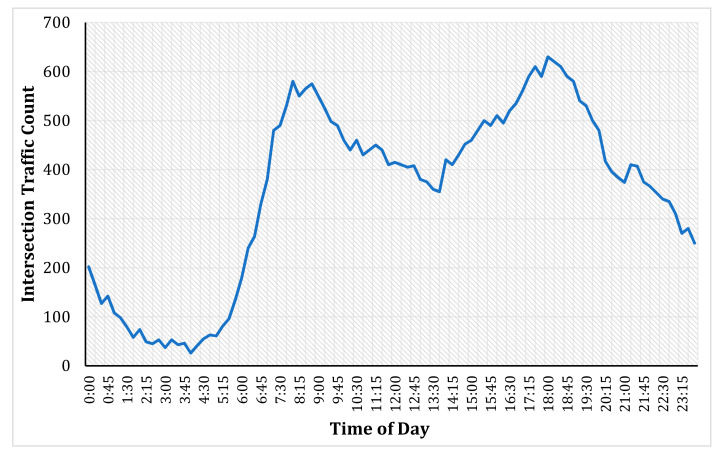
Typical intersection count profile (smooth changes).

**Figure 2:**
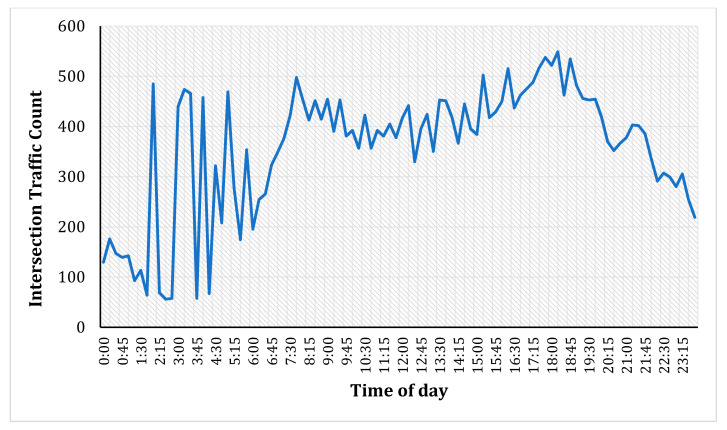
Atypical intersection count profile (severe fluctuations).

Most urban intersections show a predictable and smooth profile of traffic counts when measured at wider time-intervals (say, 5-min, 10-min, etc.). These count profiles typically consist of morning and evening peaks, mid-night off-peak, and afternoon inter-peak, as shown in Figure 1. These typical profiles show a high prognostic structure, i.e., future values can be forecasted from past values without much effort. However, the profiles can sometimes be irregular, as shown in Figure 2, which could be due to abnormal weather conditions, intersection-specific geometry, random driver behaviour, accidents, or road works in the vicinity. Commuters might experience different delays and congestion levels at the intersections than a typical day because of those conditions.

The regular occurrence of such abnormal days of traffic would result in commuters often deviating from their usual preferred (or perceived optimum) routes, and the historical information will be insufficient in picking their best route. This reduces the reliability of travel time, and only real-time information could provide significant benefits in such cases [2]. Since reliability is a crucial factor for the performance assessment of highway segments and systems, transport agencies should be able to find those irregular profiles in advance. This identification further enables them in devising efficient traffic management strategies, and proper information could be passed to commuters.

In this paper, we use the Hurst exponent method from the fractal theory to quantify fluctuations and evaluate the predictability of traffic count data at several signalised intersections in the central business district (CBD) of Sydney, Australia. Nevertheless, merely evaluating the level of predictability of data does not offer intuitions on the underlying factors. In this regard, we estimate a random-effects linear regression (RELR) model to evaluate the contributions of several factors to predictability.

First, the paper gives an overview of studies on predictability in transportation. Then, it provides a brief understanding of the Hurst exponent method and the mathematical framework of the RELR model. It is followed by a description of the study area, the data collection effort, and basic descriptive statistics of the data. Then, there is a discussion of the model results. Finally, the paper ends with a summary of findings.

## 2. Literature Review

Predictability or forecastability is considered important in decision-making in many fields, including transport. In particular, the uncertainty (or ill-predictability) of time-varying and short-term processes such as non-recurrent congestion poses severe problems for traffic authorities. Van Zuylen et al. [3] characterised the sources of ill-predictability in traffic phenomena into two categories: one due to uncertainty and incompleteness of data and models and the other due to the complexity of the processes itself [3].

There are numerous methodologies (for example, neural networks, Seasonal Autoregressive Integrated Moving Average {SARIMA}, Autoregressive Integrated Moving Average {ARIMA}, etc.) to forecast time series. However, the success of these methods relies on the structure of the particular phenomenon, whether it is easily predictable or not [4]. For instance, if the prediction obtained from a specific method is weak, but the time series contains an excellent predictive structure, one can practically conclude that the employed prediction technique is unsuitable to the task and that one should try a different technique [5]. Therefore, it is paramount to determine whether the structure of a time series is complex or easily predictable before resorting to advanced prediction techniques [6]. Nevertheless, there are only a handful of studies in the literature that evaluate the predictability of traffic entities.

Song et al. [7] used the entropy method to quantify predictability in human mobility patterns. They analysed mobile phone call data (frequency and sequence of location visits) of 50,000 individuals and found a 93% potential theoretical predictability in user mobility. Similarly, Lu et al. [8] evaluated mobile phone records of 1.9 million users in Haiti after an earthquake and quantified mobility patterns using the entropy method. They found that the upper-bound predictability is still high, at 85%. Later, Lu et al. [9] found similarly high predictability of 88% using data from mobile phone users in Ivory Coast of West Africa. Further, they used Markov chain (MC) models and found that the theoretical limit of predictability can be approached. All these studies proved that regardless of the locations travelled by users, there is high predictability in their mobility patterns.

However, mobile phone data are typically of sparse spatiotemporal resolution. Addressing this issue, Lin et al. [10] used high-resolution GPS data and still found 90% predictability in mobility sequences at an hourly sampling rate. Li et al. [11] used taxi GPS data from Shanghai and Beijing in China and found, using the entropy method, that the theoretical predictability of the location of the taxis ranges from 78 to 99%. Similarly, Wang et al. [12] used GPS data from 12,000 taxis in Beijing and found that the predictability of their movements is more than 80%. Moreover, they found that the daily traffic patterns on weekdays are of very similar predictability, despite the differences in commuter demand, and are only slightly less predictable than the weekends. Recently, Xu et al. [13] also analysed taxi data from Shanghai and found very high predictability (> 90%) of daily “travel time” time series along an expressway section at a 5-min resolution.

Lin et al. [14] used techniques including approximate entropy and the Hurst exponent to quantify the predictability of traffic volume time series of different highways in China and the USA. Further, they applied three prediction techniques (SARIMA, Support Vector Regression {SVR}, and k-nearest neighbours {k-NN}) and correlated their performance with the results from the predictability quantification methods. They found that the SVR method was suitable for non-linear datasets and SARIMA and k-NN were suitable for linear datasets. The predictability quantification methods helped them in selecting the parameters of the different prediction methods. Thus, they showed the benefits of evaluating the predictability of datasets before resorting to different prediction techniques.

The evaluation of predictability is essential for the design and improvement of prediction algorithms. All the above studies found that predictability is very high, in the range of 80–95%, in human and vehicle mobility. However, most of the studies reviewed above used the entropy method to quantify predictability, without much attention given to the other methods. The current study uses the Hurst exponent metric from the fractal theory to evaluate the predictability of intersection traffic volumes. The advantage of the Hurst exponent is that it is a numerical representation of the randomness through the history of a dynamical process. In contrast, the entropy method is independent of the temporal evolution of the dynamic process [15].

## 3. Methodology

### 3.1. Hurst Exponent

Fractal theory, introduced by Benoit Mandelbrot, is useful for studying irregularities in a time series [16]. Fractal theory deals with objects of non-integer dimension, called “Fractal Dimension” (FD), which depends on the complexity of the shape, i.e., a shape with a higher FD is more complicated or rougher than one with a lower dimension and fills more space [17]. There are various methods to estimate the FD of a time series, such as the box-counting method, Hurst exponent, and Higuchi method. However, a widely used practice for researchers is to calculate the Hurst exponent using rescaled range (or *R/S*) analysis [18,19,20]. The step-wise procedure of the standard *R/S* analysis is shown in [19,20,21].

The Hurst exponent was used by researchers to develop a predictability index (*PI*), which has the same range of 0 to 1 as the Hurst exponent. They are related, as shown in Equation (1) [22].

*PI* = 2|*H* − 0.5|) (1)

For a time series,

I. A value of *H* in the range [0.5–1] is indicative of long-term positive autocorrelation in the time series. In such cases, a high value in the series will likely be followed by another high value, i.e., the future trend is more likely to follow an established trend. For example, a very high *H* value (say *H* = 0.9) means a higher level of determinism, i.e., good predictability (*PI* = 0.8),
II. *H* values close to 0.5 indicate an entirely uncorrelated series. It means that the values in the time series are random and potentially indicating Brownian motion. The *PI*, in this case, gets closer to 0 because it becomes challenging to “precisely” predict the stochastic variations.
III. *H* value of 0 to 0.5 suggests the long-term fluctuation between high and low values in adjacent pairs of observations in the time series. A low *H* value (say *H* = 0.1) indicates a strong determinism. It is because a single high value will likely be succeeded by a low value or vice versa. Small magnitude *H* values in flow can be observed on downstream links at signalised intersections, mainly when the measurement interval is smaller than the cycle time of the signal. Due to strong determinism, the *PI* of the time series is high, even though *H* is low (*PI* = 0.8). Therefore, the *PI* for a time series is the same if the value of *H* is either 0.9 or 0.1. Furthermore, the *PI* increases when *H* approaches either 1 or 0 and decreases when it approaches 0.5.

There are many studies on the application of the Hurst exponent technique to evaluate trends in financial markets [23,24,25]. Most economics and financial time series data are “trend- reinforcing” or persistent, with *H* higher than 0.5. This technique has been used extensively in medicine [26,27], ecology [28], seismology [29], climatology [30], and hydrology [31]. In the transport domain, there have been only a few applications of the fractal theory and the Hurst exponent on real data.

Despite having several applications in many scientific fields, applications of fractal analysis of time series data have been scarcely explored in the transportation domain. In the past, researchers have applied the fractal dimension and the Hurst exponent for incident detection, short-term flow prediction, and accident warning models. Lin et al. [14] used techniques such as approximate entropy and the Hurst exponent to assess the predictability of traffic volume time series of different highways. In another study, several metrics, including the Hurst exponent and the Lyapunov exponent, were utilised to quantify the turbulence of traffic flow data of a freeway in the USA [32]. In a more recent study, it was demonstrated that high *H* values of speed could be used as a congestion indicator [21]. Moreover, the Hurst exponent has been used recently in a few studies to evaluate safety [33,34,35]. Some studies showed that for backpropagation neural networks and SARIMA models, time series with high *H* values can be predicted more accurately than those series with *H* close to 0.50 [14,19]. Further, it was observed that a smaller number of data points were required for training in SARIMA for a time series with high *H* than the ones with low *H*.

### 3.2. Random-Effects Model

Panel datasets are widely used to study the effect of spatiotemporal variations of the explanatory variables on dependent variables. In such datasets, the unobserved effects associated with a specific region will remain the same over time, thus resulting in the dependent variable being correlated over time. Similarly, there can be correlation over space because regions that are nearby may share unobserved effects. These correlations violate the assumptions of ordinary least squares (OLS) regression and misestimate the errors on the model coefficients. The random-effects (RE) and random parameters (RP) models are typically considered to account for these correlations [36,37,38,39]. In the case of the RE model, the common unobserved effects are assumed to be distributed across the spatial and temporal units according to some distribution, and shared unobserved effects are assumed to be uncorrelated with explanatory variables [40]. Therefore, the intercept term is represented by a distribution in RE models. In the case of RP models, each estimable parameter (including the intercept) of the model can vary across observations in the dataset. In this regard, the RP model can be considered as a more flexible extension of the RE model.

The RP models account for unobserved heterogeneity and offer a better fit than fixed-parameters models, yet they are time-consuming and complicated to estimate, due to the simulation-based likelihood estimation. Furthermore, the analyst must select the random parameters and their appropriate distribution. The RP approach may not necessarily improve the model, and for studies with several data points (13,468 in this study) and explanatory variables, using an RP approach can be computationally intensive due to simulation-based Halton sequences; subject to errors in specification because the modeller needs to select the variables with distributed parameters; and non-parsimonious because of the many parameters to be estimated [41,42,43,44]. Therefore, in the current study, we estimate a random-effects linear regression model (RELR) to model the theoretical predictability of traffic counts. The RELR model has the following form:

(2) $$Y_{it}=\alpha +\beta^{|}X_{it}+\varepsilon_{it}+u_{i}$$

where

$Y_{it}$ = the dependent variable, where *i* = entity and *t* = time;
α = the intercept term;
$X_{it}$ = value of the independent variable for group *i* at time *t*;
$\beta^{|}\;$= coefficient of independent variables;
$\varepsilon_{it\;}$= within entity error term, and
$u_{i}$ = between entity error term.

Furthermore, the RE model assumes that the error term of the entity is not correlated with the predictors so that the time-invariant variables can also be treated as independent variables in the regression model [45].

(3) $$\mathrm{Cov}(u_{i},\;X_{it})=0\;\mathrm{for}\;\mathrm{all}\;t,$$

(4) $$\mathrm{Exp}[u_{i}|X_{it}]\;=\;0,$$

(5) $$\mathrm{Var}[u_{i}|X_{it}]\;=\;{\sigma_{u}}^{2}\;\mathrm{and}$$

(6) $$\mathrm{Cov}[\varepsilon_{it}u_{i}|X_{it}]\;=\;0.$$

The random-effects model is a generalised regression model. It is homoscedastic, as all disturbances have variance, which is:

(7) $$\;Var\left[\varepsilon_{it}+u_{i}\right]=\sigma^{2}=\sigma_{u}{}^{2}+\sigma_{\varepsilon }{}^{2}$$

However, for given *i*, the disturbances in different periods are correlated because of their common part, *u_i_*, which is:

(8) $$Corr\left[\varepsilon_{it}+u_{i},\varepsilon_{is}+u_{i}\right]=\frac{\sigma_{u}{}^{2}}{\sigma^{2}}$$

Although there is an ML-based estimation method for the RELR model, the estimates based on generalised least squares (GLS) are efficient [45]. This study uses the GLS estimator.

## 4. Study Area and Data Collection

### 4.1. Study Area

The CBD area of Sydney, Australia is the focus area for this study. Sydney is the largest city in Australia and the eighth largest in the southern hemisphere, with an estimated population of 5 million. The city is famous for its sporting events, New Year fireworks, three-week lighting festival in May and June, and several other special events that run throughout the year. Further, the busy, modern, and vibrant lifestyle attracts millions of tourists from across the world. The CBD of Sydney employs 13% of the Sydney region’s workforce and generates a quarter of the Sydney economy [46]. Public transport is the primary mode for commuters during weekdays. Traffic congestion is a major problem in the city, being ranked 1st among the cities in Australia and Oceania [47]. The morning and evening peak hours are especially notorious for congestion, increasing travel times by around 67% compared to the free-flow conditions.

### 4.2. Data

The data for this study were obtained from Sydney Coordinated Adaptive Traffic System (SCATS) intersection counts in the Sydney CBD. SCATS is a fully adaptive urban traffic control system that optimises traffic flow at intersections. It gathers traffic count data in real time at each intersection and then makes incremental adjustments to traffic signal timings based on the variations of traffic counts at the intersections [48]. Therefore, SCATS is an ‘‘on-line’’ algorithm, in which the designed control strategy ‘‘matches’’ the current traffic conditions to the ‘‘best’’ pre-calculated off-line timing plan [49]. Its self-calibrating software minimises manual intervention, which can result in substantial operational cost savings. Loop detectors are located on all the lanes at the stop line, which record the counts of vehicles for every pre-specified time interval.

**Figure 3:**
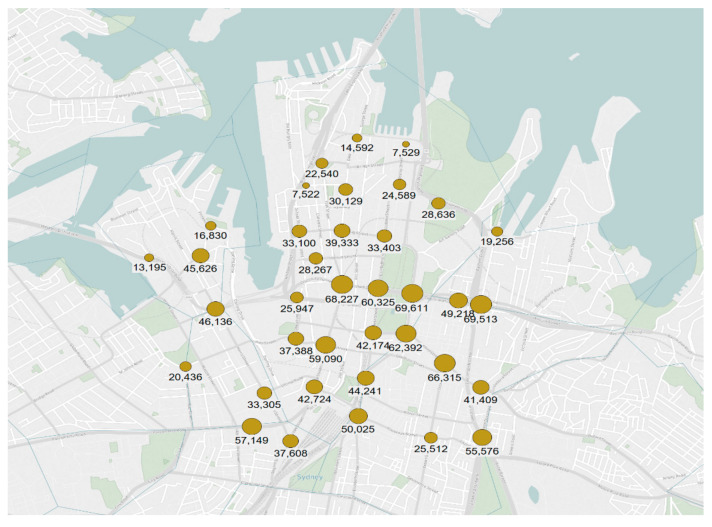
Layout of the analysed intersections in the Sydney Central Business District.

We collected traffic count data at a 5-min resolution at around 180 signalised intersections in and around the Sydney CBD, from 15th October 2014 to 13th October 2015, translating to 364 days of observations. However, after careful examination for missing and erroneous data, we selected 37 intersections for the analysis. Figure 3 shows the layout of these 37 intersections along with their average annual daily traffic (AADT). Although the SCATS interface outputs count data at each detector, we considered the aggregated data for the analysis. The geometric features of the intersections vary with respect to the number of approaches, number of bus stops in the vicinity, number of lanes, and number of parking lanes. We estimated the Hurst exponent and the predictability index (using Equation (1)) for every day at each intersection. In total, there are 13,468 (37 signals × 364 days) observations of *H* and *PI*.

**Figure 4:**
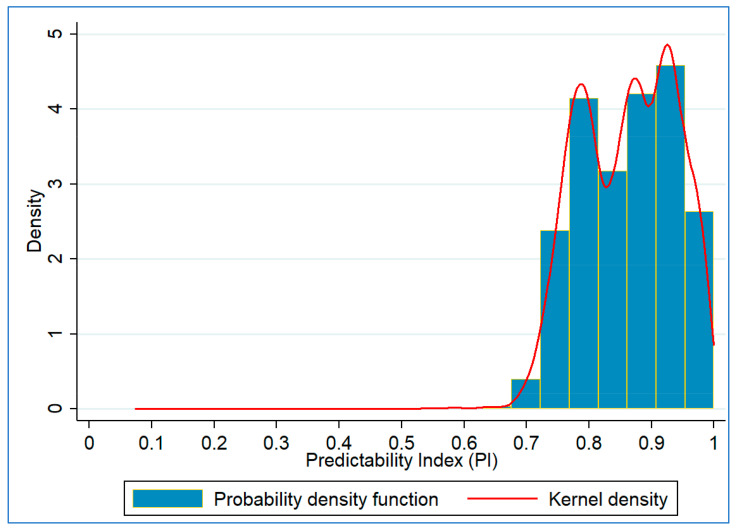
Probability density function and kernel density plots of predictability index (PI).

Figure 4 shows the Probability Density Function (PDF) and kernel density plots of *PI*. A highly sizeable portion of the *PI* observations are close to 1, indicating a highly predictable trend at most intersections on most days. However, a non-trivial proportion of observations exist with slightly lower predictability (*PI* < 0.8). Furthermore, the kernel density estimation shows three modes of the *PI*. This paper investigates the influence of various factors behind such low/high predictability.

## 5. Data Analysis

### 5.1. Preliminary Analysis

**Figure 5:**
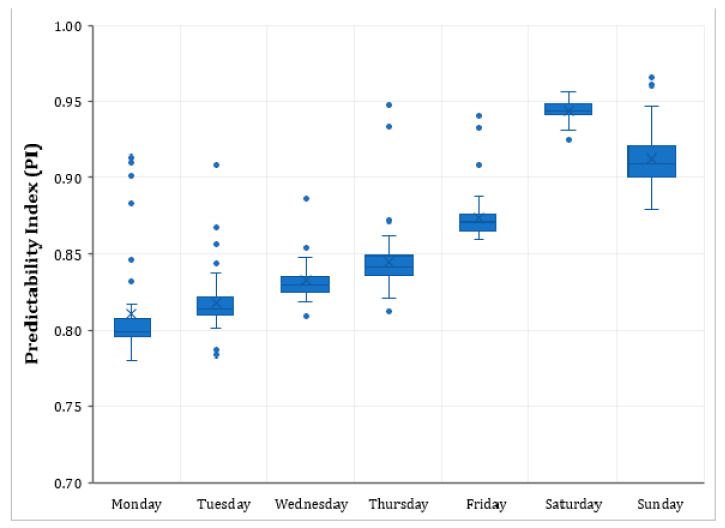
Variation in average *PI* (of all intersections) with the day of the week.

It is surmised that the day of the week will have a profound impact on predictability. Figure 5 shows the variation in the average *PI* of all the intersections with the day of the week. The weekends (Saturdays and Sundays) tend to have higher predictability, compared to the regular weekdays. Moreover, the variation of *PI* is low on weekends. It is also seen that the predictability is low on Mondays and gradually increases as the week goes on.

We performed a one-way ANOVA test for the average *PI* of the day of the week. This test showed that the differences are statistically significant (F (6, 13461) = 1246.53, *p*-value < 0.001). Further, Tukey’s posthoc Honest Significant Difference (HSD) test showed that the mean *PI* of each day of the week is statically different to every other day, implying a strong influence of the day of the week on intersection count predictability. However, there are several special event days (There were several special events during the data collection period that could have significantly affected the traffic movement in the CBD. Cricket matches (World Cup and Big Bash League), football league games, New Year Eve’s fireworks, marathon runs, Vivid lights festival, and Mardi Gras parade are some of these events. As may be seen in Table 1, there were 33 of them during the study period. Because of the varying nature of individual events, it is difficult to classify which event is special. In this study, all the events that had attendance of at least 10,000 people were considered “special events”.), public holidays, days with heavy rain, during the analysis period, which should be accounted for while modelling predictability. Additionally, the role of intersection geometry and the presence of bus stops and parking cannot be ignored. The following section presents the results of the RELR model, which considers the above-specified factors.

### 5.2. Model Results

Table 1 shows the descriptive statistics of all the potential variables considered in the model estimation. We arranged the dataset in a panel data format, consisting of daily estimated theoretical predictability indices of traffic counts at 37 signalised intersections. Thus, the dataset is a strongly balanced panel with 13,468 observations, including 37 panels (all of them have the same number of observations, i.e., 364). We collated data of the weather, public holidays, special events, and other relevant variables from various sources for the model estimation. Then, we classified the variables into two types, namely space-varying and time-varying, that vary across the intersections and days, respectively.

In determining the best model specification for the sample, we estimated several RELR models by changing the variables. The variables that were significant at *p* = 0.10 were considered. After each model specification, we performed multicollinearity diagnostics by estimating the variance-covariance matrix and correlation matrix of the estimated coefficients. Table 2 shows the best model in terms of goodness-of-fit out of all the RELR model specifications explored. The table shows the coefficients of the variables, along with robust standard errors and *z* and *p* values.

Furthermore, we conducted the Breusch–Pagan Lagrange multiplier test (Breusch and Pagan 1980). Based on the test, we rejected the null hypothesis that variance across entities is zero, indicating that there are significant differences across panels units. In other words, a random-effects model is better than a simple pooled OLS regression. The final model presented in Table 2 has only the significant variables. The model has an *R*-square value of 0.61.

There are two categorical variables in the model, namely the day of the week and the parking indicator, which have “Monday” and “No parking” as base categories, respectively. The coefficients of other categories are estimated with respect to these categories. The model has a significant constant of 0.755, suggesting that the mean *PI* on Mondays at intersections with parking restrictions is 0.755 when all the other variables are considered zero.

## 6. Discussion

The day of the week has a strong influence on the *PI*. The predictability of all other days is significantly higher than that of Mondays. Notably, the weekends are highly predictable, with mean predictability increasing by 10–14%, while the other variables are held constant. Moreover, congestion likely occurs on the weekdays, and so, it becomes easy to reroute on urban roads rather than, say, motorways, resulting in more fluctuations and, therefore, low predictability on weekdays.

Earlier studies also found that the type of day can have an enormous impact on the highway traffic pattern. For example, Rakha and Van Aerde [2] found that traffic flows on core weekdays will be different from Mondays, Fridays, Saturdays, and Sundays. Similarly, Weijermars and Berkum [50] classified working days into (1) Mondays, (2) core weekdays, (3) Fridays, and (4) days within the vacation period. In these studies, the core weekdays were found to have similar traffic flow patterns. Even in the current study, the predictability varies within the range of 4% from Monday to Thursdays but is significantly higher on other days of the week.

Days with special events are found to have better predictability. One can theorise that the throughput profile rises fairly linearly before the start of the event and then drops similarly after the event, resulting in high *PI*.

Public holidays increase the *PI* of traffic counts by 0.07 when all other variables are held constant. The traffic profile on public holidays tends to be similar to that of Sundays [51], which could be a reason for the similar predictability levels.

Rainfall is found to have a negative impact on the *PI*. While a light shower may not significantly reduce the predictability index, rainfall of more than 20 mm per day could reduce it by 0.01 when other factors are held constant. Travel behaviour could significantly change when there is significant rainfall. Heavy rains could persuade the otherwise public transit commuters to travel by car or even cancel their trips and work from home. Past research suggested the importance of accounting for rainfall in short-term traffic predictions [52] as rainfall was found to reduce travel demand and average speeds, particularly on weekends [53]. Recreational trips were found to have more sensitivity to weather changes [54]. Therefore, even the theoretical predictability index of traffic count reduces due to heavy rainfall.

The temperature has a small yet positive and significant effect on the *PI*. A 10 °C increase in temperature could increase *PI* by 0.01, given that all other variables are held constant.

Table 2 shows that the intersection geometry also has some role in predictability. The intersections with at least three parking lanes (i.e., on at least three approaches) are found to have higher *PI* than the ones with either no parking or up to two lanes. On-street parking is limited in the CBD, and therefore, the intersections near the roads with more parking spaces receive a continuous stream of vehicles throughout the day. Therefore, such intersections tend to have less fluctuations and thus higher *PI* of traffic counts.

## 7. Conclusions

Traffic counts at intersections are consistent and repetitive, on the one hand, and yet can be variable and less predictable on the other hand, in which, at any given time, unusual circumstances such as crashes and adverse weather, erratic driver behaviour, etc., can dramatically change the conditions of road traffic. These anomalies can create congestion and uncertainty in the transport networks and, therefore, from an operational standpoint, it is crucial to detect as early as possible potential irregular traffic patterns.

We used the Hurst exponent method to quantify fluctuations in traffic count data at several intersections in the Sydney CBD. *PI* is typically upwards of 0.80 (i.e., 80%) for most of the days at most signals. This finding is in line with the earlier studies that found that human mobility is highly predictable, with the upper range of 80 to 95%.

Although techniques such as entropy, fractal dimension, and Hurst exponent are useful in quantifying the predictability of time series, they do not offer intuitions on what makes a time series hard to predict. Understanding the various aspects leading to high/low complexity in the dataset is critical for better prediction results and the choice of prediction methods. In this regard, we estimated a random-effects linear regression model to identify the variables that significantly influence the predictability of traffic counts. The statistical analysis revealed that the count predictability is strongly associated with the day of the week, with lowest on Mondays and highest on Saturdays. Public holidays and special event days are found to have a positive impact on the *PI*. Rainfall has an adverse effect, but the temperature has a small yet significant positive effect. Finally, the intersections with parking on more than two approaches are more predictable than the ones with no parking or parking on up to two approaches.

The predictability index calculated in this study is only a theoretical one and independent of the prediction method, which can be slightly higher or lower. However, it can aid in the development of prediction methods in choosing the right parameters. It is inappropriate to claim superiority of a prediction technique over other techniques without evaluating the complexity of the time series data. The proposed technique would be genuinely superior if the *PI* of the time series data is low, but still, the predictions are correct. On the other hand, good predictions for a dataset with already high *PI* by “sophisticated” methods may not be of much help.

Predictability of traffic counts could be evaluated to identify the intersections that are more reliable than others. Thus, predictability could be used as one of the performance measures of intersections such as vehicle throughput, delays, queues, and fuel emissions.

Intersection count predictability could be affected by the geographical location of the intersection. For example, intersections in a suburb may have an intermittent throughput of vehicles than, say, CBD, where a continuous stream of vehicles exists. The driving habits of the populace, the number of parking manoeuvres, the percentage of heavy vehicles, pedestrian movement, and side friction could influence the predictability. These aspects should be considered in future studies. Furthermore, there could be spatial autocorrelation present in the dataset because of the close proximity of the intersections. Statistical techniques accounting for unobserved heterogeneity, such as a random parameter model or latent class model, could be used in the future.

## Figures and Tables

**Table 1:** Descriptive statistics of all the considered variables.

Type of Day	Count	Type of Variable	% of Observations (Categorical Variables)	For Continuous Variables			
				Mean	Std. Dev.	Max	Min
**Day of week**							
**Monday**	52	TV	14.29				
**Tuesday**	52	TV	14.29				
**Wednesday**	52	TV	14.29				
**Thursday**	52	TV	14.29				
**Friday**	52	TV	14.29				
**Saturday**	52	TV	14.29				
**Sunday**	52	TV	14.29				
**Type of day**							
**Public holiday**	11	TV	3.02				
**Special event day**	33	TV	9.07				
**Season**							
**Spring**	90	TV	24.73				
**Autumn**	92	TV	25.27				
**Winter**	92	TV	25.27				
**Summer**	90	TV	24.73				
**Rainfall (mm)**	364	TV		3.65	10.27	116.02	0
**Temperature (°C)**	364	TV		19.03	4.44	29.2	9.6
**Average Annual Daily Traffic (AADT)**	37	SV		36,360	15,772	64,189	7237
**Lanes**	37	SV		9.54	3.33	16	3
**≤8 lanes**	7	SV	18.92				
**8–12 lanes**	23	SV	62.16				
**>12 lanes**	7	SV	18.92				
**Approaches**	37	SV		3.43	0.59	4	1
**1, 2**	2	SV	5.41				
**3**	17	SV	45.95				
**4**	18	SV	48.64				
**Parking lanes**	37	SV		1.35	1.07	4	0
**No parking**	10	SV	27.03				
**1–2 lanes**	22	SV	59.46				
**3–4 lanes**	5	SV	13.51				
**Bus stops**	37	SV		1.27	1.22	5	0
**No bus stop**	12	SV	32.43				
**1–2 bus stops**	20	SV	54.05				
**3,4, and 5 bus stops**	5	SV	13.51				
**Crashes**	37	SV		18.16	14.70	72	1

TV—time-varying. SV—space-varying.

**Table 2:** Results of the Random Effects Linear Regression (RELR) model.

Variable	Coefficient	Robust Std. Error	Z	*p* >|z|
**Day of the week**				
**Monday**	Base			
**Tuesday**	0.0135	0.0027	4.98	<0.01
**Wednesday**	0.0276	0.0037	7.41	<0.01
**Thursday**	0.0367	0.0046	7.90	<0.01
**Friday**	0.0642	0.0064	10.02	<0.01
**Saturday**	0.1336	0.0086	15.52	<0.01
**Sunday**	0.1035	0.0063	16.55	<0.01
**Type of day**				
**Special event day**	0.0119	0.0018	6.47	<0.01
**Public holiday**	0.0705	0.0057	12.42	<0.01
**Weather**				
**Rainfall (in 10 mm)**	−0.0049	0.0007	−6.43	<0.01
**Temperature (in 10 °C)**	0.0093	0.0021	4.49	<0.01
**Parking**				
**No parking**	Base			
**1–2 lanes**	Insignificant			
**3–4 lanes**	0.0680	0.0209	3.25	<0.01
**Constant**	0.7675	0.0128	60.03	
**Sigma_u**	0.0404			
**Sigma_e**	0.0422			
**Rho**	0.4781			

## Data Availability

Restrictions apply to the availability of data used in this study. Data was obtained from Transport for New South Wales, Sydney, Australia.
